# 1211. Impact of Gram-Negative Rod Bacteremia Rapid Diagnostic Testing and Real-Time Clinical Staff Pharmacist Review

**DOI:** 10.1093/ofid/ofad500.1051

**Published:** 2023-11-27

**Authors:** Abby Kremer, Alison Orvin

**Affiliations:** WakeMed Health and Hospitals, Raleigh, North Carolina; WakeMed Health and Hospitals, Raleigh, North Carolina

## Abstract

**Background:**

Although Rapid Diagnostic Testing (RDT) enables optimization of antimicrobial therapy, there is no consensus on how to integrate utilization into clinical practice. At our institution, the BIOFIRE® Blood Culture Identification 2 (BCID2) RDT was implemented in September 2021 in conjunction with real-time clinical staff pharmacist result notification and review. The objective of this study was to determine the impact of RDT plus real-time clinical staff pharmacist review on the management of gram-negative rod (GNR) bacteremia.

**Methods:**

This retrospective, matched cohort study included patients with a positive blood culture for a GNR on the BCID2 panel from September 2020 to August 2021 (historical) and October 2021 to September 2022 (interventional). Exclusion criteria were polymicrobial bacteremia, discrepant RDT results from traditional culture, 24-hour mortality, and comfort care or not admitted at the time of RDT result. Patients were randomly screened to include 120 patients in each group and matched based on age, pathogen, and resistance mechanism. The primary endpoint was time from Gram stain to appropriate antibiotic therapy. Secondary endpoints included time to first pathogen-directed change in therapy, days of anti-pseudomonal therapy, length of stay (LOS), and inpatient mortality.

**Results:**

The study population was predominantly female (61%) and the majority were over 70 years old (55%). *Escherichia coli* was isolated in 71% of patients with extended-spectrum beta-lactamase-producing organisms isolated in 8%. The median time to appropriate therapy was 0 hours for both groups, indicating patients were on appropriate therapy at the time of Gram stain result (p=0.28). There was a statistically significant decrease in time to first pathogen-directed change in therapy (40 vs. 11 hours; p< 0.01). There was no difference in days of anti-pseudomonal therapy, LOS, or inpatient mortality between groups.

**Conclusion:**

Implementation of RDT plus real-time clinical staff pharmacist intervention did not impact time to appropriate therapy in patients with GNR bacteremia but did significantly reduce time to pathogen-directed antibiotic changes. RDT plus real-time clinical staff pharmacist intervention is a practical method of utilizing technology to improve patient care.

**Disclosures:**

**All Authors**: No reported disclosures

